# The medicinal mushroom *Ganoderma lucidum* attenuates UV-induced skin carcinogenesis and immunosuppression

**DOI:** 10.1371/journal.pone.0265615

**Published:** 2022-03-21

**Authors:** Ayaz Shahid, Matthew Huang, Mandy Liu, Md Abdullah Shamim, Cyrus Parsa, Robert Orlando, Ying Huang

**Affiliations:** 1 Department of Pharmaceutical Sciences, College of Pharmacy, Western University of Health Sciences, Pomona, California, United States of America; 2 College of Osteopathic Medicine of the Pacific, Western University of Health Sciences, Pomona, California, United States of America; 3 Department of Pathology, Beverly Hospital, Montebello, California, United States of America; Annamalai University, INDIA

## Abstract

The medicinal mushroom *Ganoderma lucidum* is traditionally used for treating multiple diseases, including cancer. This study examined skin cancer preventive activity of a commercial product containing spore and fruiting body in 30:8 ratio (GLSF). Extracts of GLSF and spore component (GLS) were prepared using artificial gastrointestinal juice and examined on JB6 cells. GLSF and GLS dose-dependently inhibited epidermal growth factor-induced JB6 transformation at non-toxic concentrations. SKH-1 mice which were fed with diets containing GLSF (1.25%), GLS (0.99%) or the fruiting body (GLF) (0.26%) were exposed to chronic low-dose ultraviolet (UV) radiation to assess their effects on skin carcinogenesis. GLSF, but not GLS or GLF, reduced skin tumor incidence and multiplicity. In non-tumor skin tissues of mice, GLSF attenuated UV-induced epidermal thickening, expression of Ki-67, COX-2 and NF-κB, while in tumor tissues, GLSF increased expression of CD8 and Granzyme B. To examine the effects of GLSF on UV-induced immunosuppression, mice which were fed with GLSF were evaluated for the contact hypersensitivity (CHS) response to dinitrofluorobenzene (DNFB). GLSF significantly reversed UV-mediated suppression of DNFB-induced CHS by increasing CD8+ and decreasing CD4+ and FoxP3+ T-cells in mouse ears. Therefore, GLSF prevents skin cancer probably via attenuating UV-induced immunosuppression.

## Introduction

Skin cancer is a major type of cancer with a well-known association with environmental factor–solar ultraviolet (UV) radiation. Overexposure to UV radiation, particularly in the mid-wavelength range (UVB, 280–320 nm), is the most significant risk factor for the development of non-melanoma skin cancers (NMSC), including basal cell and squamous cell carcinomas (BCC and SCC), which originate from keratinocytes. In contrast to many other tumor types, NMSC incidence is increasing at an alarming rate in the US—3 to 4 million cases of BCC and 1 million cases of SCC annually [1–3]. Chemoprevention, i.e., using a pharmacological approach to block or reverse the initiation, promotion and progression of cancer, represents a very attractive strategy for the control of NMSC.

UV radiation not only induces DNA damage and skin inflammation, but also creates an immunosuppressive microenvironment in which premalignant cells are able to grow into tumors [4]. Only when mutations and immunosuppression coexist, it is possible for skin tumors to occur [5]. Supporting this notion, organ transplant recipients who receive long-term immunosuppressant treatment are at increased risk for skin cancer [6]: for BCC, there is a 10-fold increased risk in organ transplant recipients, while the risk of developing SCC is increased by 65–250 times. UV-irradiated skin is unable to elicit sensitization to contact allergens by development of immune tolerance to them [7]. UV-irradiated skin is also unable to reject syngeneic UV-induced tumors, although the same tumors are strongly rejected when transplanted into non-UV-exposed syngeneic mice [8]. Therefore, overcoming UV-induced immunosuppression could be an important strategy for skin cancer prevention.

Since the target population for cancer chemoprevention is healthy individuals, pharmacological agents that can be used for the preventive purposes should be both effective and safe. Therefore, natural products have become a very attractive source for developing cancer preventive agents. *Ganoderma lucidum* (GL), commonly known as Reishi or Lingzhi, is a medicinal mushroom recorded in the American Herbal Pharmacopoeia (2000) as one of the most popular and highly effective herbal medicines and is frequently used as an ingredient in dietary supplements. It has been used for thousands of years for the prevention and treatment of many diseases due to a wide spectrum of actions, including immunomodulatory, anti-inflammatory, antioxidant, and antitumor properties [9]. The most important and widely studied pharmacologically active compounds in GL are polysaccharides and triterpenoids [10, 11].

The present study examined the chemopreventive efficacy and potential mechanisms for GL on skin carcinogenesis. GLS and GLF are commercial products derived from the broken spore or fruiting body of GL, respectively. GLSF is a relatively new GLS-derived product containing GLS and GLF in a 30:8 ratio. The present study demonstrated that GLSF has skin cancer chemopreventive activity both *in vitro* and *in vivo*. In a chronic UV induced skin carcinogenesis mouse model, GLSF, but not GLS or GLF, prevented UV-induced skin carcinogenesis *in-vivo*. To explore a possible mechanism of GLSF’s anticancer effects, an established model of contact hypersensitivity (CHS) in mice was used to evaluate the effects of the dietary GLSF on the immunosuppressive effects of UV. These results led us to conclude that this specific product derived from GL containing both spore and fruiting body may have a novel function in skin cancer prevention. Although this study used murine skin carcinogenesis models, these data may support further clinical investigations of GLSF on other types of cancer.

## Materials and methods

### Preparation of GLSF extracts

A single batch of a commercial product manufactured by Beijing Tong Ren Tang Chinese Medicine Co. (Hong Kong, China), named as GLSF, containing a mixture of the spore (GLS) and fruiting body of GL (GLF) (30:8 ratio in weight), was used throughout the present study. The artificial gastrointestinal juice was prepared according to reported methods [12, 13]. In brief, the gastric extracts were prepared by mixing 5 grams of GLSF or GLS powder in 50 mL of artificial gastric juice in 37°C shaker for 1 hour, then 50 mL of artificial intestinal juice was added and incubated at 37°C shaker for additional 5 hours. The mixture was centrifuged at room temperature for 15 minutes at 4,700 g, and the supernatant was collected. The solution was then neutralized to pH 7.0 using 0.2 N NaOH. The extract was filtered using Whatman filter paper No. 42, lyophilized, and stored at -20°C. An established HPLC-DAD fingerprint method based on 11 chemical markers has been used for identity determination of GLSF, GLS, and GLF [14].

### Cell line and cell culture

The JB6 CI 41-5a (also named JB6 P+) is a mouse epidermal cell line purchased from American Type Culture Collection (ATCC, Manassas, VA) sensitive to promotion by growth factors or environmental stressors, including EGF. The JB6 P+ cells were cultured in Eagle’s minimum essential medium (EMEM) containing 4% heat-inactivated fetal bovine serum and 1% penicillin/streptomycin and incubated at 37°C in 5% CO_2_/95% air.

### UV light source

UV lamps emitting UVC (100–280 nm; 2% of total energy), UVA (320–400 nm; 37% of total energy), UVB (280–320 nm; 54% of total energy), and visible light (400–450 nm; 7% of total energy; catalog nos., #95-0042-08 and #95-0043-13, UVP) were used to irradiate in both in vitro and in vivo experiments. UVX Radiometer (#97-0015-02, UVP) coupled with a sensor with a calibration point of 310 nm (UVX-31, #97-0016-04, UVP) was used to measure stable power output (mW/cm^2^). The exposure time was determined using the following formula: dose (mJ/cm^2^) = exposure time (s)*output intensity (mW/cm^2^). Quality control of the lamps and exposure time were calculated and monitored before using each of the lamps to account for power output changes.

### JB6 anchorage independent soft-agar assay

The JB6 P+ cell line (neonatal BALB/c epidermal cells) is sensitive to transformation. The soft agar assay supports cells to grow in 3-dimension, mimicking in vivo cell growth. The process of this assay has been described previously [15]. In brief, In a 96-well tissue culture plate, 2x10^3^ JB6 P+ cells were mixed with 0.33% agar and suspended on top of a solidified bottom layer containing 0.5% agar. Nobel Agar (Sigma-Aldrich) was prepared in PBS, autoclaved, and stored at 4°C. EGF (10 ng/mL) was used to promote and stimulate the anchorage-independent growth of JB6 P+ cells. The GLSF or GLS extracts were mixed with EGF and added to both agar layers. Plates were incubated at 37°C with 5% CO_2_/95% air for 10–14 days. Colonies larger than 10 cells were counted manually under a microscope. The anchorage-independent soft agar assay can be used to identify chemopreventive activities of GLSF or GLS extracts by accessing their ability to suppress cell transformation of JB6 P+ induced by EGF.

### Cell proliferation assay

96-well plates were seeded with 3,000 to 4,000 cells per well and allowed to attach overnight. Cells were treated with test compounds for 72 hours and incubated at 37°C in 5% CO_2_/95% air. According to the manufacturer’s protocol, cell viability was determined using Sulforhodamine B (SRB) assay (Sigma) according to manufacturer’s protocol.

### Rodent dietary supplement of GLS, GLF, and GLSF

To evaluate the cancer preventive effects of GLSF in comparison with GLS and GLF in vivo, the test agents were homogeneously blended into the AIN 93G diet by Animal Specialties (Hubbard, OR). The formulations included 1.25% GLSF, 0.26% GLF, or 0.99% GLS. The drug dose calculation was based on an average daily intake of 4 grams per mouse. Food consumption for each group was monitored weekly during the course of the study and confirmed that the dose of GLSF, GLF and GLS was approximately 2.0 g/kg, 0.42 and 1.58 g/kg, respectively.

### Skin carcinogenesis studies in mice

All animal studies were carried out in accordance with the recommendations in the Guide for the Care and Use of Laboratory Animals of the National Institutes of Health and approved by the Western University of Health Sciences Institutional Animal Care and Use Committee (Pomona, CA). Five-week-old female SKH-1 mice (Charles River, Wilmington, MA) were randomly divided into five groups (n = 12). For a diet-based drug regimen study, Groups 1 and 2 were fed with the standard diet AIN 93G, while groups 3, 4 and 5 were fed with diet containing GLS, GLF and GLSF. Mice were switched to modified diet 1 week after arrival. 4 weeks after starting the modified diet, the mice were irradiated with gradually increasing levels of UV three times a week for 27 weeks with an initial dose of 50 mJ/cm^2^ that is increased each week by 25 mJ/cm^2^ to 150 mJ/cm^2^, which is continued for the duration of the experiment. During the UV exposure, mice roamed freely in acrylic cages on a rotating platform with rotational placement ensuring consistent and equal dorsal distribution of UV irradiation. Tumors of at least 1 mm in diameter were counted and measured with a caliper weekly. The tumor volume was calculated according to the formula: (width)^2^ x length/2. At the end of the study, animals were anesthetized with isoflurane until respiration ceased, and all efforts were made to minimize suffering. Tumorous and non-tumorous skin samples were excised and fixed in 10% formalin for pathological analysis.

### Contact hypersensitivity (CHS) assay

The UV radiation-induced suppression of the immune system in mice was assessed using the CHS model, as described previously [16]. In brief, the SKH-1 hairless mice (females) from the breeding cohort were randomly distributed into 5 groups (6–8 weeks). The mice were pretreated with a modified diet containing GLS, GLF, GLSF or control diet for 4 weeks. Mice were irradiated with a single dose of UV (336 mJ/cm^2^) on the back. During exposure, the ear skin of mice is protected by covering with black electric tape. 24 hours after UV exposure, the mice were sensitized with freshly prepared 2,4-dinitro-1-fluorobenzene (DNFB) (Sigma) (25 uL, 0.5% in acetone:olive oil 4:1, vol/vol) on UV treated back skin. Seven days later, the mice were challenged with DNFB (10 ul, 0.5%) on the right ear skin (dorsal side only) (the other ear treated with vehicle). 24 hours after the challenge, the mice were euthanized and 5 mm punch biopsies were obtained from the ears and weighed before fixation in 10% formalin. Previous studies have demonstrated that measurement of weights of ear punch biopsies correlated with measurement of ear thickness with calipers [16]. We prefer this method due to greater reproducibility in our data. Non-UV irradiated mice that received the same dose of DNFB serves as a positive control. Non-UV-irradiated mice that received only ear challenge serve as a negative control.

### Histopathological analysis and immunohistochemical staining for detection of NF-kB, COX-2, Ki-67, CD8, CD4, Granzyme B, and FoxP3

For the carcinogenesis study, the non-tumorous and tumorous skin tissues, and the CHS study, the ears were excised, and all the tissues were fixed in 10% neutral formalin and embedded in paraffin. The paraffin-embedded tissues were then de-paraffinized using xylene and ethanol. To analyze histology, the de-paraffinized sections were stained with hematoxylin and eosin (H&E). In IHC, for antigen retrieval, sections were boiled in antigen retrieval Buffer (cat no. ab93678) for 20 minutes. Different expression of proteins in skin tissue was determined using the Vectastain Elite ABC universal plus kit (PK-8200). In brief, sections were incubated with bloxall endogenous enzyme blocking solution for 10 min to quenching of endogenous peroxidase activity and then rinsed three times (5 min each) with TBST (0.05% Tween-20). Blocking solution was applied for 20 minutes; then sections were incubated with diluted polyclonal antibodies, anti-COX-2 (1:200, Cell Signaling Technology), anti- NF-kB (p65) (1:400, Cell Signaling Technology), Ki-67 (1:400, Cell Signaling Technology), CD8 (1:400, Cell Signaling Technology), CD4 (1:100, Cell Signaling Technology), Granzyme B (1:125, Cell Signaling Technology) and FoxP3 (1:400, Cell Signaling Technology) overnight at 4°C in a humid chamber [For more information about antibodies see S1 Table]. Further processing was performed according to the instructions of the Detection System. Scoring the images from IHC analysis was carried out using a semi-quantitative scoring system. Ki-67 was scored by counting the positively stained cells in 4 to 5 fields at various locations along the skin section to obtain the average for each mouse skin (n = 3 mice for long-term UV study). To quantitate CD8, CD4, Granzyme B, FoxP3 COX-2, and NF-kB staining, an expression index was calculated by the extent of staining multiplied by the intensity of staining. For the extent of staining, the following system was used: 0, no staining, 1 is < 25% of cells positive, 2 is >25% and ≤50% of cells positive, and 3 is >50% of cells positive. For staining intensity, the following system was used: 0 is no staining, 1 is faint staining, 2 is moderate staining, and 3 is strong staining. Skin sections were evaluated at 20x or 40x magnification using a Leica DM750 LED Biological Microscope.

### Statistical analysis

Data are expressed as mean +/- standard error or standard deviation and was analyzed using GraphPad Prism software. One-way ANOVA analysis with Dunnett’s multiple comparison test or student t test was used. For all statistical analysis, mean was indicated to be statistically different when *P* < 0.05.

## Results

### Effects of GLSF and GLS extracts on EGF-induced neoplastic transformation of JB6 P+ cells

**Fig 1:**
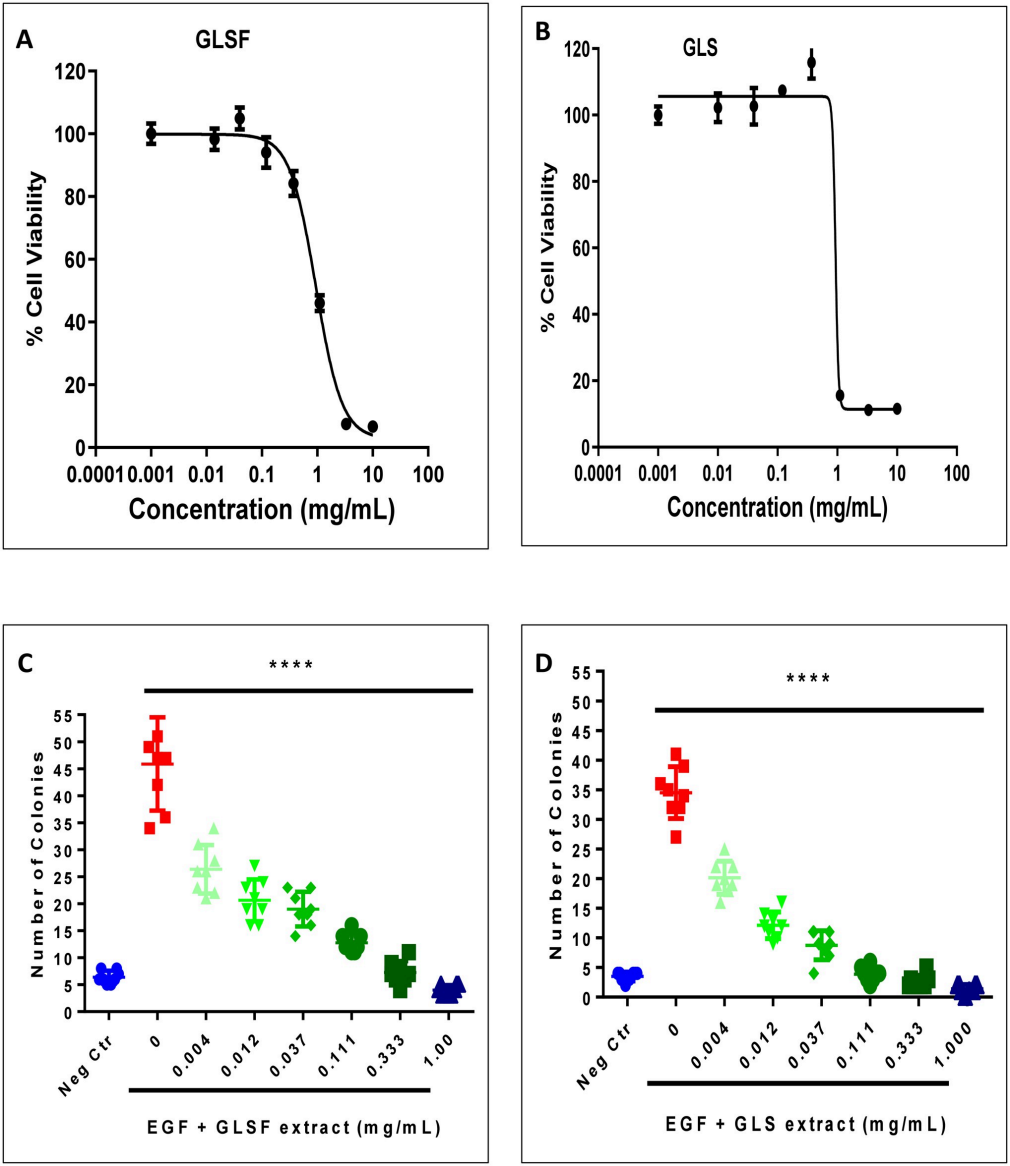
Effects of GLSF and GLS gastrointestinal juice extracts on cell viability and transformation in JB6 P+ cell line. (A) GLSF extract was examined for cytotoxicity and growth inhibitory activity on monolayer culture of JB6 P+ cells using SRB assay. Estimated IC_50_ value was 1.0 mg/mL. (B) GLS extract was examined for cytotoxicity and growth inhibitory activity on monolayer culture of JB6 P+ cells using SRB assay. Estimated IC_50_ value was 0.75 mg/mL. (C) GLSF extract at various concentrations was examined for its anti-transformation activity, i.e., suppressing cell transformation of JB6 induced by EGF (10 ng/mL) using soft agar assay. Estimated IC_50_ value was 0.0085 mg/mL. (D) GLS extract at various concentrations was examined for its anti-transformation activity on soft agar. Estimated IC_50_ value was 0.0075 mg/mL. ****: *P* < 0.0001 as determined by ANOVA and Dunnett’s post hoc test.

Since signaling through EGF and its receptor EGFR promotes skin cancer, EGF was used to promote the transformation of mouse epidermal cells JB6 P+ (the tumor promoter sensitive subline). EGF (10 ng/mL) consistently induced anchorage-independent colony formation as expected [17]. As the colony formation in soft agar is dependent on cell viability, we first conducted a SRB cytotoxicity assay in JB6 P+ cells growing in a monolayer to evaluate the effects of these extracts on growth inhibition for this specific cell type. The GLSF and GLS extracts showed comparable in vitro effects on cell proliferation. For both extracts, SRB assay showed dose-dependent growth inhibitory activity (IC_50_ was estimated as 1.0 mg/mL) (Fig 1A and 1B). Next, the extracts at the dose range of 0.004 to 1.0 mg/mL were examined on EGF-induced JB6 P+ cell transformation. The data showed that both GLSF and GLS inhibited transformation (i.e., soft agar colony formation) in a dose-dependent manner (Fig 1C and 1D) (*P* < 0.0001 for all doses examined). The IC_50_ for the transformation inhibitory activity of GLSF and GLS was estimated at 0.0085 and 0.0075 mg/mL, respectively. Thus, GLSF and GLS inhibited epidermal malignant transformation at non-toxic concentrations. Since transformation of JB6 P+ is an assay predictive for *in vivo* cancer preventive activity [18], these data suggest that GL products, both GLSF and GLS, are able to prevent skin cancer.

### Effect of GLSF and its components GLS and GLF on chronic UV-induced skin tumor formation in SKH-1 mice

**Fig 2:**
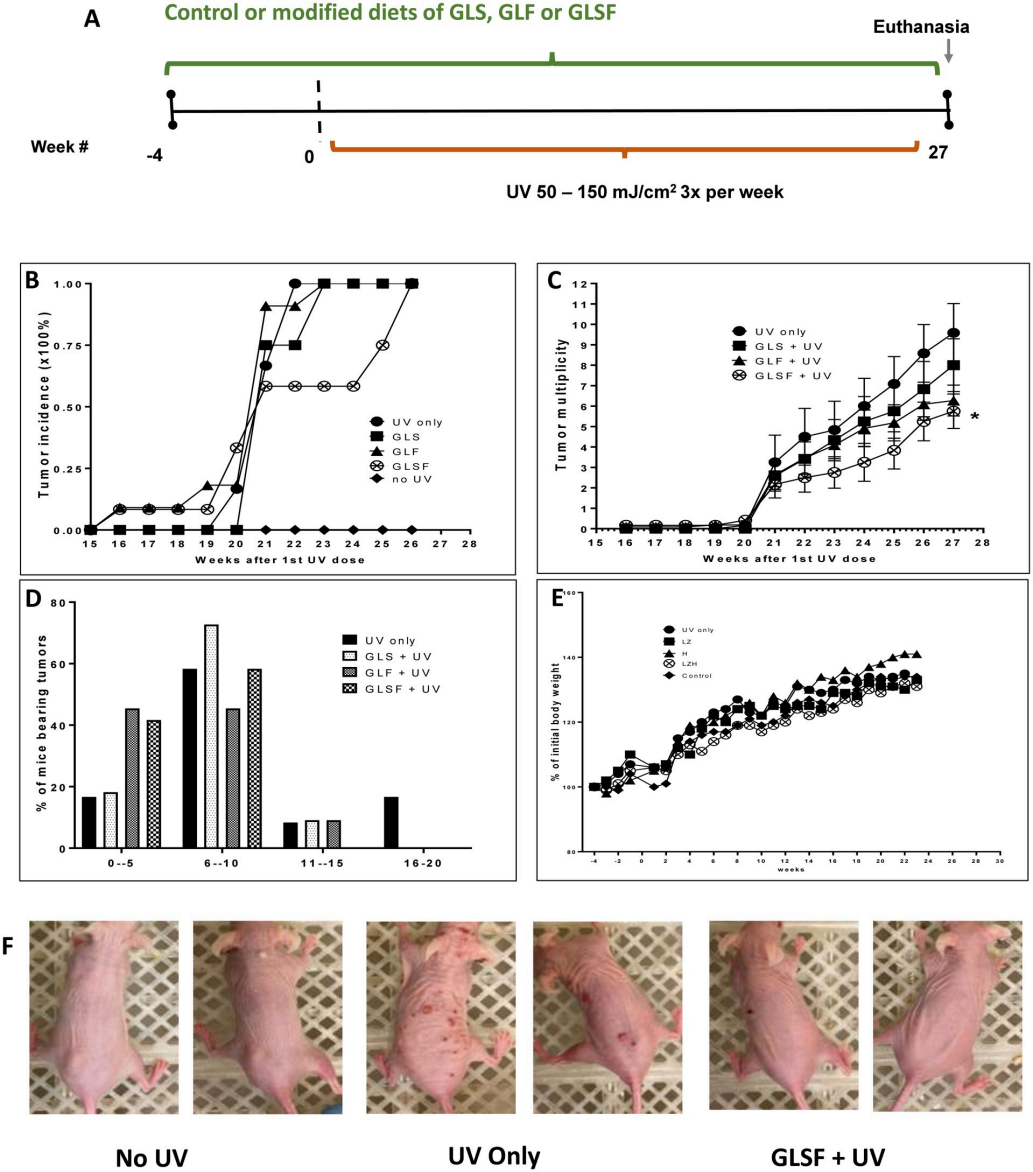
Effects of GLS, GLF and GLSF on development of skin tumor in SKH-1 hairless mice. (A) Experimental design. The mice were pre-treated with control diet or modified diets containing GLS, GLF or GLSF for 4 weeks before UV exposure was initiated. Then the mice were exposed to UV radiation of increasing doses three times per week for 27 weeks. (B) The percentage incidence of mice bearing tumors. (C) Tumor multiplicity (number of tumors per mouse). *: *P* < 0.05 as determined by t test. (D) Percentage of mice bearing tumors in each treatment group. (E) Average body weight in each treatment group (% of initial body weight). (F) Representative photograph of control mice without UV exposure, mice with UV only, and mice treated with GLSF and UV.

The chemopreventive activity of dietary GLSF, in comparison with GLS and GSF, was evaluated in SKH-1 hairless mice which were exposed to chronic UV radiation. The mice were pre-treated with the control diet (AIN93G) or a modified diet containing GLS (0.99%, estimated dose 1.58 mg/kg), GLF (0.26%, estimated dose 0.42 mg/kg), or GLSF (1.25%, estimated dose 2.0 mg/kg, containing a mixture of GLS and GLF in 30:8 ratio) for four weeks before the first UV exposure (Fig 2A). The UV dose was initiated from 50 mJ/cm^2^ and increased by 25 mJ/cm^2^ every week until the maximal dose of 150 mJ/cm^2^ was achieved and maintained for the remaining course of the experiment. The first visible tumor appeared in GLF, GLSF and GLS groups at the week 16, 16 and 21 after UV exposure started, respectively, while the first visible tumor appeared at the week 20 in the UV only group (Fig 2B). However, after 20 weeks, a drastic increase in the number of tumors was observed in all groups. The tumor incidence data indicate delayed occurrence of skin tumor in GLSF treatment group as compared to the control. By the end of the week 26, although 100% of mice in the GLSF group had tumors, the treatment with GLSF demonstrated a significant reduction of the tumor multiplicity compared with the control group (*P* < 0.05; Fig 2C). Although treatment with GLS or GLF showed a trend reducing the tumor multiplicity (Fig 2C) (*P* > 0.05), their tumor incidence data overlap with the UV only group (Fig 2B). The % of mice bearing larger tumor is also reduced by treating with the GSLF diet, because in GLSF group, none of the mice bearing more than 10 tumors (Fig 2D). The body weight data for all groups overlapped, indicating that the treatments did not cause significant toxicity (Fig 2E). Representative images of mouse tumors are taken at week 23 after UV initiation (Fig 2F). At this time point (week 23), the tumor incidence was 100% in the control, GLS and GLF groups, while in GLSF group there were only 58% tumor-bearing mice (Fig 2B). These data indicate that dietary GLSF may prevent skin carcinogenesis induced by UV. Such activity may be attributed to a combinational or synergistic effect of GLS and GLF, since the individual components showed modest effects on the same experimental model.

### Histopathological and immunohistochemical staining analysis of non-tumor skin from GLSF treated mice

**Fig 3:**
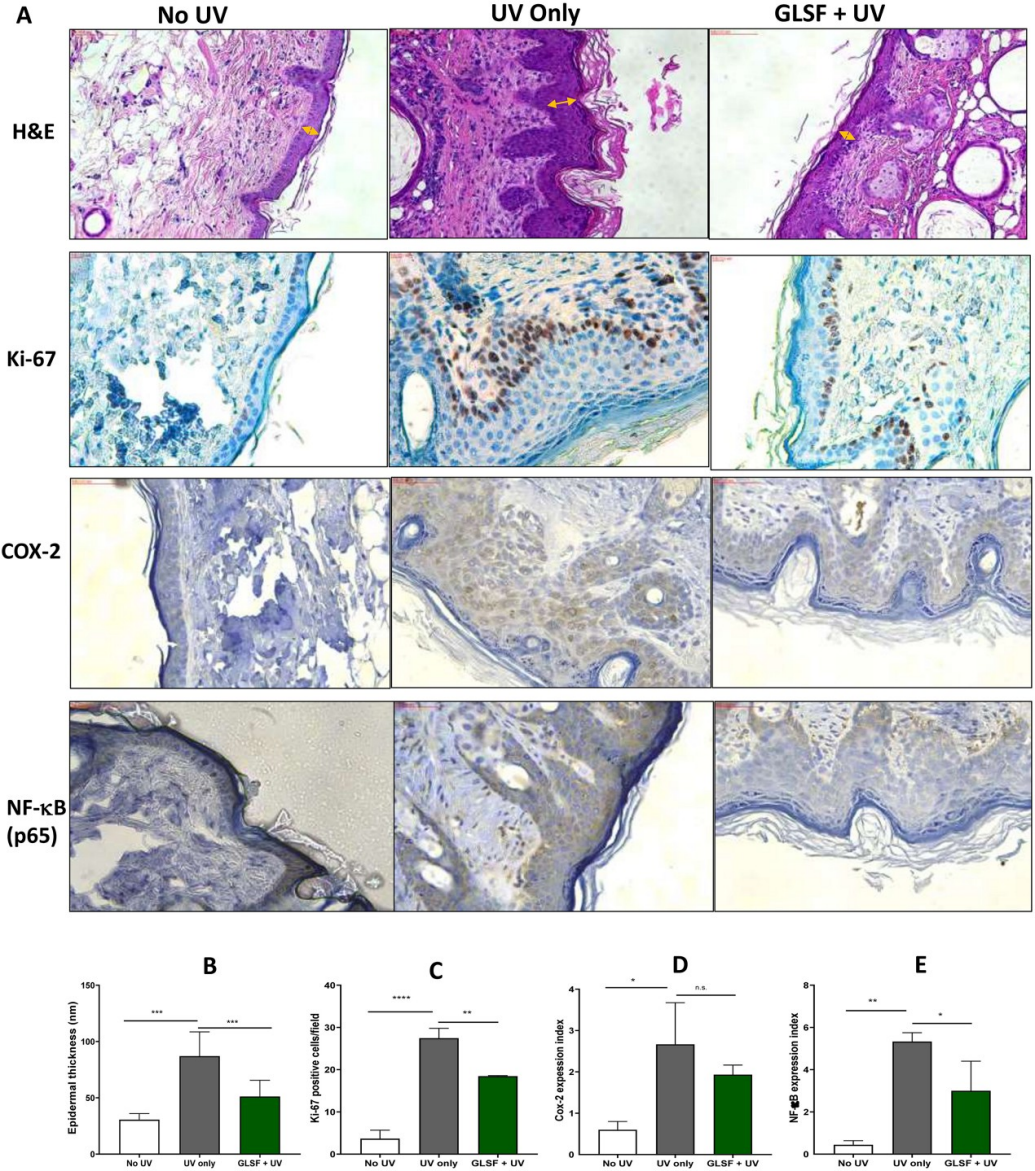
Effects of GLSF and UV on histology and inflammatory markers in non-tumor skin in SKH-1 mice. (A) Representative microphotographs of skin of mice treated with UV, with or without GLSF treatment stained with H&E (40X) and IHC of Ki-67, COX-2 and NF-κB (p65) (40X). For IHC, brown color indicates specific immunostaining of Ki-67, NF-κB and COX-2 and light blue color indicates hematoxylin staining. (B) Measurement of epidermal thickness in H&E-stained slides of mice. The thickness was measured 10 times at various locations along with the epidermis and averaged to obtain a single skin sample data (n = 5). (C) Quantification of the number of Ki-67 positive cells, which were counted in three randomly selected fields and averaged to obtain a single skin sample data (n = 5). (D) and (E) The expression index was used to quantify the extent and intensity of COX-2 and NF-κB-p65 expression in each skin sample (n = 5). An ANOVA followed by a Tukey-Kramer multiple-comparison post hoc test was used to assess statistical differences at p < 0.05.

Fig 3A portrays the histological investigation of the non-tumorous skin sections of control and GLSF treatment groups in the UV exposure experiment. H&E staining of the skin tissues from negative control (no UV) mice illustrated normal epidermal and dermal architecture with normal thickness. The skin sections from UV treated mice revealed strongly increased epidermal thickness and formation of skin tumors *in situ*. The GLSF‐treated animals showed a partial reversal of the histological damages induced by UV and significantly reduced the epidermal thickening (Fig 3A and 3B).

Consistent with the epidermal thickness data (Fig 3A), based on immunohistochemical (IHC) expression analysis, the Ki-67 immuno-positive cells, which are mainly present in the basal layers, was significantly increased in UV treated group as compared to untreated controls (Fig 3A and 3C). Decreased number of Ki-67 positive cells was found in the GLSF treated group as compared only UV treated group, indicating that GLSF treatment prevents tumor development via inhibiting cell proliferation (Fig 3C).

Genes controlling skin inflammation are under the transcriptional control of the redox sensitive transcription factor NF-κB [19]. NF-κB has been shown to play a role in induction of organ toxicities such as skin, liver, kidney and pancreas by oxidative stress including UV radiation. To evaluate the effect of GLSF on UV-induced chronic inflammation in skin tissue, we investigated the levels of total expression of NF-kB p65 protein and COX-2 expression by IHC as shown in Fig 3A. Brown color clearly indicates increased immunostaining of NF-κB (p65) as well as COX-2 in UV-exposed groups. Treatment with GLSF reduced the immunostaining of NF-κB and COX-2 in comparison with UV only group (*P* < 0.05 for p65, *P* > 0.05 for COX-2) (Fig 3D and 3E). These data demonstrated that the preventive effects of dietary supplements of GLSF on UV-induced skin tumor development may be partially due to the protective effects of GLSF against UV-mediated inflammation.

### Histopathological and immunohistochemical staining analysis of skin tumors from GLSF treated mice

**Fig 4:**
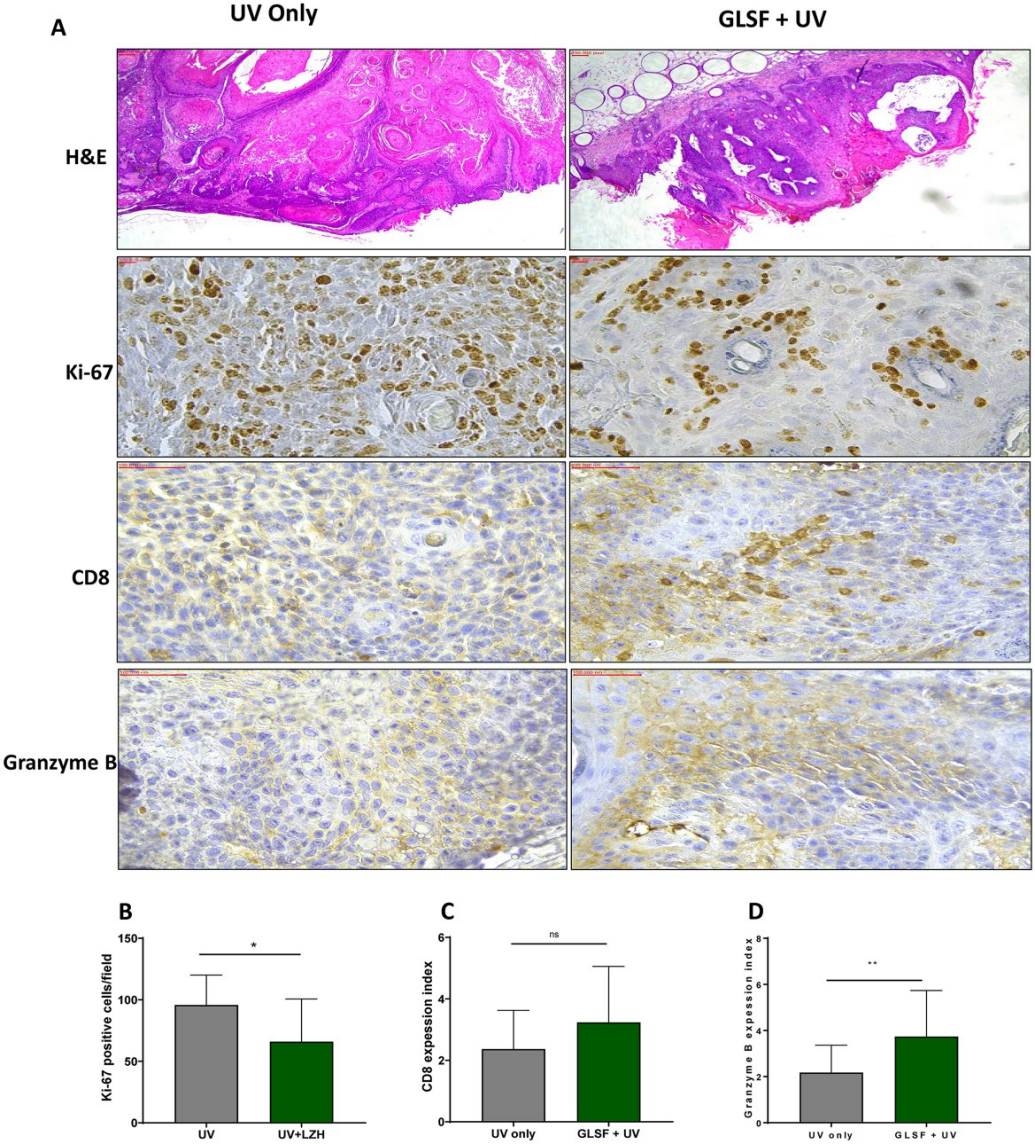
Effects of GLSF and UV on histology and expression of Ki-67, CD8 and Granzyme B in skin tumor in SKH-1 mice. (A) Histology of tumor tissues showing SCC and papilloma in control and GLSF treatment groups (H&E staining, 4x) and IHC analysis of Ki-67 (40X), CD8 (40X) and Granzyme B (40X) treated with UV with or without GLSF. (B) Quantification of the number of Ki-67 positive cells, which were counted in three randomly selected fields and averaged to obtain a single skin sample data (n = 5). (C) The expression index was used to quantify the extent and intensity of CD8 and (D) Granzyme B expression in tumor samples obtained from control or GLSF groups (n = 5). An unpaired t test was used to assess statistical differences. *: *P* < 0.05, **: *P* < 0.01. ns: not significant.

Within each treatment group of control diet or GLSF diet, 10 largest tumors were excised and fixed in formalin for H&E and IHC analysis. H&E staining of tumors from both groups of mice showed mixture of SCC and benign papilloma (Fig 4A). The IHC analysis of Ki-67 showed less number of Ki-67 positive tumors in GLSF treated mice (Fig 4A and 4B) (*P* < 0.05). Interestingly, IHC analysis indicates that the expression index of CD8 and Granzyme B in GLSF treated tumors were stronger than tumors from UV only group (Fig 4A, 4C and 4D) (*P* > 0.05 for CD8; *P* < 0.05 for Granzyme B). These IHC data suggest that GLSF may change the local tumor T-lymphocyte infiltration to a pattern that may decrease tumor progression or facilitate a direct cytotoxic effect. These data are consistent with the known effects of GL on immunomodulation.

### Effects of GLSF on UV-induced suppression of the CHS response in mice

**Fig 5:**
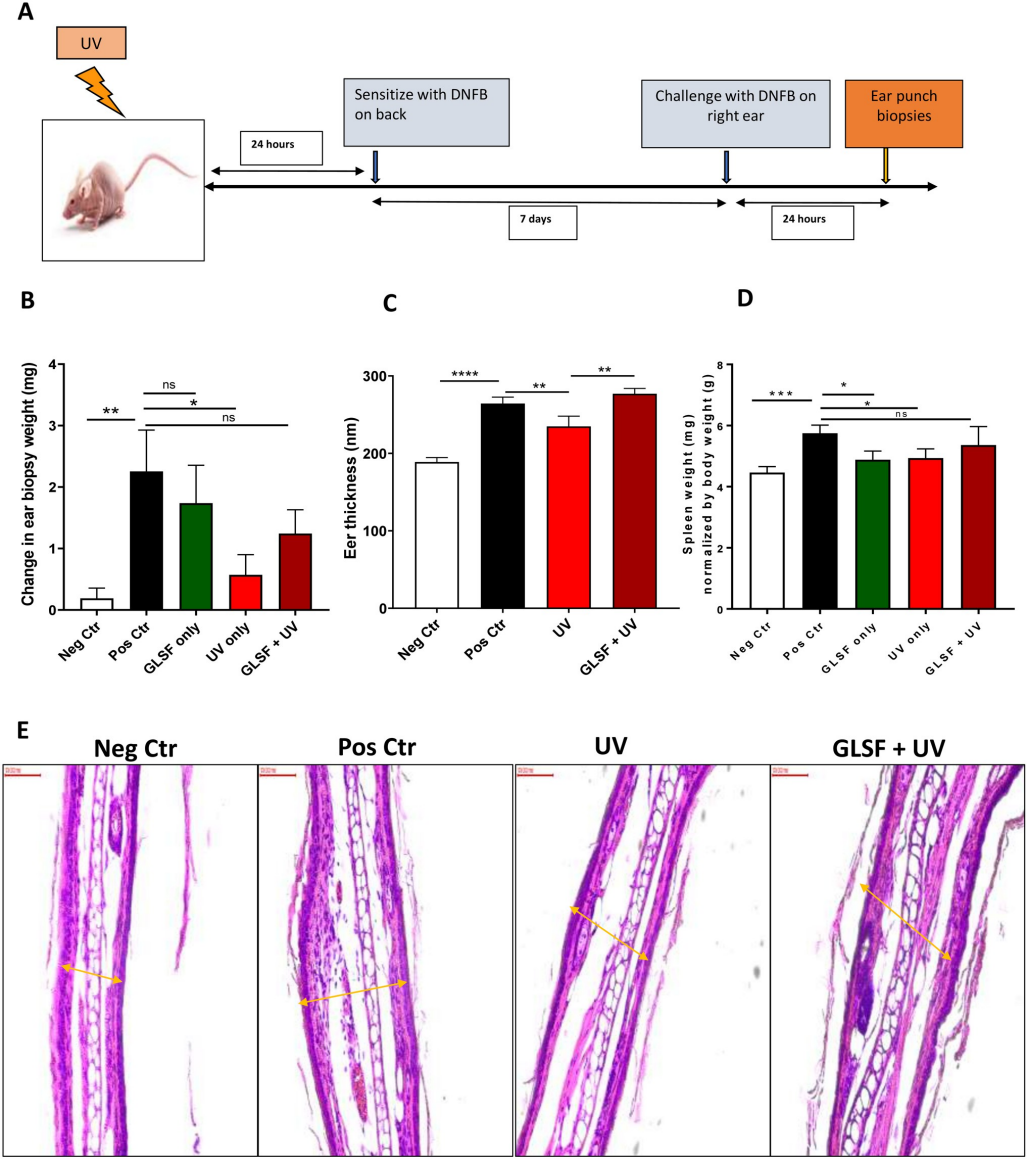
Effects of GLSF on UV induced suppression of CHS in mice. (A) Scheme of the experimental protocol. SKH-1 mice were treated with UV radiation before sensitized to DNFB and elicitation reactions (challenge) were performed on the dorsal ears. (B) At 24 hours after the challenge, 5 mm punch biopsies were obtained from the ears and weighted. The data listed are the mean +/- SE of difference in ear punch biopsy weight (right-left) from n = 5 mice. (C) The weight of the spleen is normalized by body weight. (D) The ear thickness was also measured on the H&E-stained ear sections. (E) Representative microphotographs of mouse ear punch biopsy stained with H&E (20X). *: *P* < 0.05. **: *P* < 0.01. ***: *P* < 0.001. ****: *P* < 0.0001. ns: not significant by an unpaired t test.

GL has been suggested with an immunostimulant activity [20]. As dietary GLSF prevents UV-induced skin tumor formation and expression of T-cell activation markers CD8 and Granzyme B, we next examined whether treating mice with GLSF has any protective effects against UV-induced immunosuppression (Fig 5A). Immunosuppression induced by UV has been demonstrated by a classical contact hypersensitivity (CHS) response to contact allergens such as dinitrofluorobenzene (DNFB) [21]. In the absence of treatment with GLSF, the CHS response in terms of ear biopsy weight difference was significantly lower in those mice that were UV-irradiated than those mice that were non-irradiated, confirming the immunosuppressive effect of the UV radiation in these mice. A single dose of UV radiation (336 mJ/cm^2^) suppressed the CHS reaction to 25% of non-UV-treated positive control (*P* < 0.05) (Fig 5B). The mice which were pretreated with a modified diet containing GLSF (estimated dose 2.0 g/kg) for 4 weeks before UV exposure showed a trend of reversing UV-induced CHS suppression to 55% of the positive control. GLSF alone did not affect the ability of the mice to generate a CHS response to DNFB in the absence of UV irradiation (Fig 5B). The mouse ear sections were imaged with H&E staining. The measurement of ear thickness indicated that UV-induced suppression of CHS was significantly lower in the groups of mice treated with GLSF and UV irradiation than in the mice that were not treated with GLSF (Fig 5C and 5E). These results demonstrated that dietary treatment with GLSF can protect mice from UV-induced immunosuppression. Interestingly, the spleen weight data from each treatment group indicate that DNFB induced a significant increase of spleen weight in mice, which was reduced by treatment of GLSF or UV alone (P < 0.05) (Fig 5D). However, combined treatment of GLSF and UV radiation attenuated reduced spleen weight so that there was no statistically significant change between the GLSF+UV group versus the positive control group. These data, for the first time, indicate that GLSF blocks UV-induced harmful effects on cutaneous immunity. Since the exposure of the skin to UV radiation inhibits mice from making an immune defense against their tumors, the cancer preventive effects of GLSF may be partially attributed to its activity against UV-induced immunosuppression.

**Fig 6:**
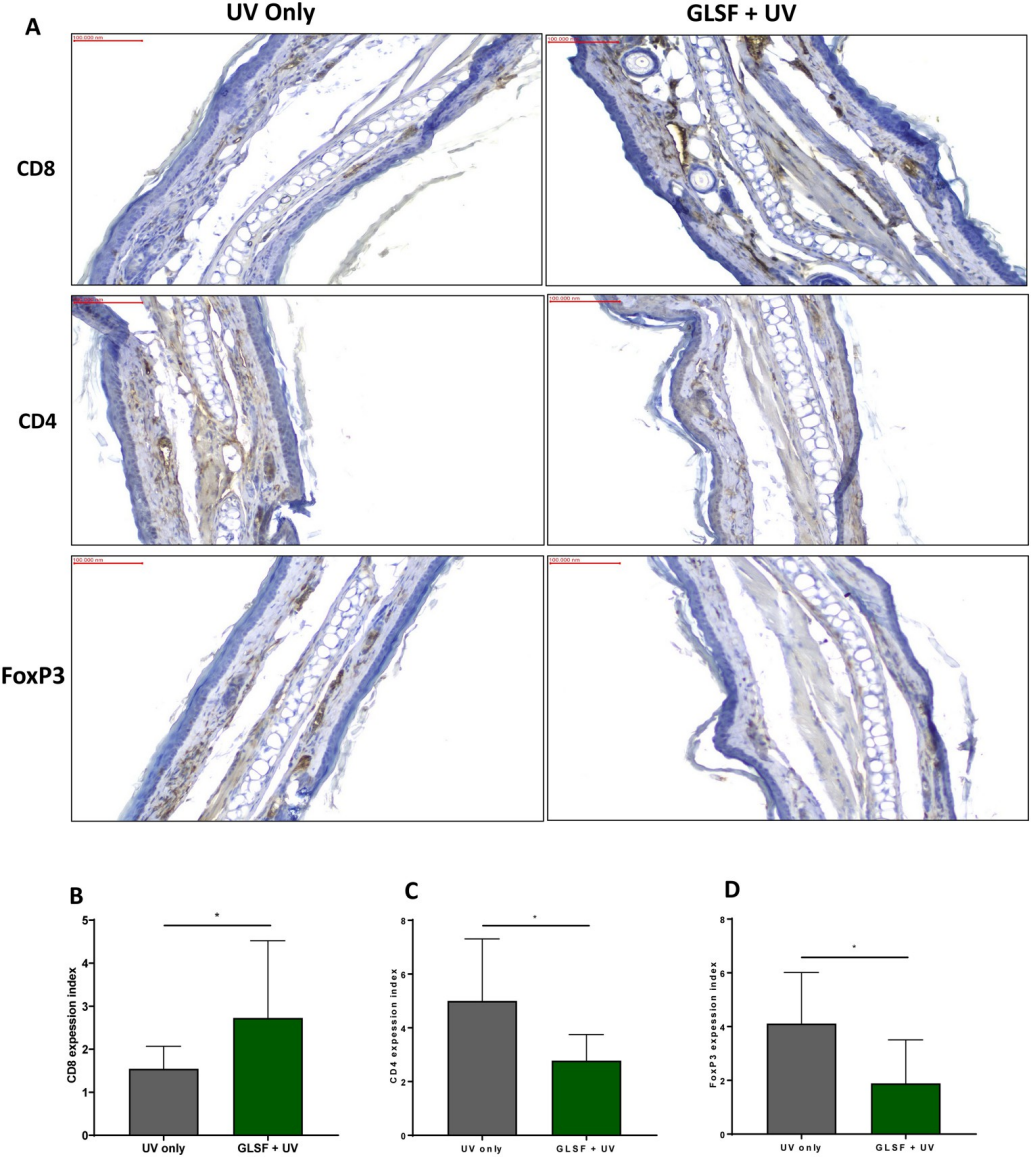
Immunohistochemical analysis of the T-lymphocyte infiltrate during CHS to DNFB. SKH-1 mice were treated with UV radiation before sensitized to DNFB and elicitation reactions (challenge) were performed on the dorsal ears. At 24 hours after the challenge, 5 mm punch biopsies were obtained from the ears. (A) Representative microphotographs of mouse ear punch biopsy stained with primary antibodies of CD8, CD4 or FoxP3 for IHC analysis (20x magnification). (B) The expression index was used to quantify the extent and intensity of CD8, CD4 (C) and FoxP3 (D) expression in ear samples obtained from UV only or GLSF + UV groups (n = 5). An unpaired t test was used to assess statistical differences. *: *P* < 0.05.

Previous studies have shown that CHS to DNFB is mediated by CD8+ effector cells. Using IHC, we have found that in GLSF/UV treated mice, more CD8+ cells were recruited into the ear skin at the site of DNFB challenge, compared with mice treated with UV alone (Fig 6A and 6B). As a marker for regulatory T (Treg) cells, CD4+ cells showed the opposite trend, in that the number of CD4+ cells were decreased in GLSF treated mice skin compared with mice treated with UV alone (Fig 6A and 6C). Consistently, the staining of FoxP3, another marker for activated Treg cells, was also decreased in GLSF treated mice (Fig 6A and 6D). These data were consistent with the notion that UV radiation suppressed the immune system by UV-induced increase of Treg cells [22] and that GLSF is able to reduce the activity of Treg.

## Discussion

In recent years, traditional herbal medicines have gained more interest as a complementary and alternative approach of cancer therapy because of their insignificant side effects on the human body. *Ganoderma lucidum* (GL), as an important natural product derived from the medicinal mushroom, draws attention since it enhances human health and increases longevity [23]. It has exhibited significant anti-tumor effects against different types of tumors [24–26] via multiple mechanisms involving anti-proliferation, pro-apoptosis, anti-metastasis, and anti-angiogenesis [24]. In the present study, we evaluated the cancer preventive and anti-immunosuppression properties of GL using established skin carcinogenesis models.

The non-tumorous murine epidermal JB6 P+ cells and UV-induced murine skin carcinogenesis are well-established models to investigate the mechanism of skin carcinogenesis and to identify natural or synthetic chemopreventive agents. We first used the *in vitro* assay of JB6 P+ transformation induced by the tumor promoter EGF to examine the effects of gastrointestinal extracts of GLSF and its component GLS. Since the data showed a highly potent anti-transformation activity for both extracts, *in vivo* studies were also conducted in SKH-1 mice. The tumors formed in UV-irradiated SKH-1 mice imitate human skin SCC formation and reflect clearly defined tumor formation stages [27]. Dietary GLSF treatment decreased the tumor incidence and reduced the average numbers of tumors per mouse, indicating a protective activity against carcinogenesis. Furthermore, epidermal thickness and histopathological observations of the non-tumor skin clearly indicate that GLSF supplementation reversed the chronic inflammation caused by UV and showed nearly habitual architecture, which might contribute to the decrease in tumor formation, implying the antitumor action of GLSF. However, GLS and GLF did not show the same degree of prevention as GLSF, indicating that the combination of these two components may synergistically contribute to the activity of GLSF.

The Ki-67 is a marker usually used to estimate cellular proliferation in different types of cancers, including skin cancer [28, 29]. It is a protein that consists of two isoforms having molecular weights of 345 and 395 kDa, and its expression level persists high through all active phases of the cell cycle, including G1, S, G2, and M, but low in the resting phase (G0). Therefore it is considered a clinically important index to show the proliferation capacity of tissues [30, 31]. The expression of Ki-67 is also correlated with the proliferative activity of tumor cell populations and is commonly used as a marker for tumor aggressiveness [32–34]. A reduction in the expression of this marker is linked with a response to cancer treatment [35]. The prognostic importance for Ki-67 IHC staining has been examined in numerous cancer studies, including skin cancer [29, 36–40]. In our study, GLSF significantly ameliorated Ki-67-positive cells in the non-tumor and tumor skin tissue compared with the UV only group (Figs 3A and 4A). Our study is consistent with the previous report in which GL treatment decrease Ki-67 cell expression [41].

In our study, we found that GLSF treatment decreased the expression of COX-2 and NF-kB significantly as compared to the only UV group (Fig 3A). Our results indicate that inflammation is a major target for GLSF and previous studies validate our data [42, 43]. Inflammation is the body’s response to tissue damage and coordinates with cellular transformation and the immune system to help repair injured tissue [44, 45]. Different types of mediators are produced by immune cells during the inflammation play an important role in making homeostasis balance [44, 46, 47]. However, in chronic inflammation, overproduction of immune mediators can disrupt this homeostasis balance and secrete excessive pro-inflammatory agents, including prostaglandin E2 (PGE_2_), via up-regulating the Cox-2 activity, which leads to inflammation mediated diseases, including cancer [48]. Cox-2 and its product PGE2 play an important role in the skin’s response to UV radiation which has been demonstrated by Cox-2 knockout mice [49]. Cox-2 inhibitors such as nonsteroidal anti-inflammatory drugs (NSAIDS) have shown chemopreventive activity against UV-induced skin carcinogenesis [50]. Thus, our data indicate GLSF prevents skin cancer via targeting COX-2 expression. Although the exact mechanism is unknown, since GL is a non-toxic dietary supplement, it offers advantages of improved safety in comparison with nonsteroidal anti-inflammatory drugs.

Activation of Nuclear factor-κB (NF-κB) has been linked to various cellular processes in cancer, including inflammation and proliferation [51]. It is a transcription factor that governs a wide array of genes associated with various immune and inflammatory responses, including inflammation of the skin [52]. The inactive NF-κB exists as a heterodimer of the p65 and p50 subunits in the cytoplasm, which are bound to the inhibitory protein IκB [52]. Activation of NF-κB can be induced directly by UV or indirectly by pro-inflammatory cytokines such as tumor necrosis factor and IL-1. Therefore, drugs that inhibit NF-κB activity have been useful as a chemopreventive agent in various cancers [53]. The p65 subunit of NF-κB is essential for skin carcinogenesis in mice since the loss of p65 in the keratinocytes prevented both SCC tumor initiation and tumor promotion [54]. GLSF treatment significantly attenuated NF-κB expression in UV exposed mice, suggesting it have potent anti-inflammatory activity.

UV-induced immunosuppression has been linked to the development of photocarcinogenesis. We investigated the efficacy of GSLF on UV-induced immunosuppression using local contact hypersensitivity assay in mice. Contact hypersensitivity (CHS) is an immune cell-dependent T cell-mediated response elicited by various sensitization chemicals [55]. A hypersensitivity in mice and humans is activated by Th1/T cytotoxic-1 effector cells and downregulated by Th2/T regulatory (Treg) CD4+ T cells [56–58]. The role of GLSF in UVB-induced immune suppression of CHS is unknown. These mechanisms are of significant clinical importance as UV-induced immunosuppression has been associated as a risk factor for nonmelanoma skin cancers. To test the effect of GLSF in UV-induced immunosuppression, we treated GLSF to mice one month before UV exposure, and the results showed that GLSF reversed the immunosuppression effect of UV effectively (Fig 6). Further studies are needed to identify the active components in GLSF that are responsible for such cancer preventive and immunomodulatory activity.

In conclusion, our data strongly provide evidence that dietary supplements of GLSF may have preventive effects against UV-induced skin tumor development, which may be partly attributed to GLSF’s activity on modulation of cutaneous immunity.

## Supporting information

S1 Table Antibodies used in the manuscript with company name, vendor name, catalog no. and dilution factors. (DOCX)
