# Supplementary material for: The medicinal mushroom Ganoderma lucidum attenuates UV-induced skin carcinogenesis and immunosuppression
Source: PLoS One. 2022 Mar 21;17(3):e0265615. doi: 10.1371/journal.pone.0265615 (PMC8936451; doi:10.1371/journal.pone.0265615)
Supplement: S1 Table — (DOCX) [file pone.0265615.s001.docx]

S1 Table. Antibodies used in the manuscript with name, vendor name, catalog no. and dilution factors

|  | **Name** | **Vender** | **Catalog** | **Dilution Factor** |
| --- | --- | --- | --- | --- |
| 1 | Cox-2 | Cell Signaling Technology | 12282S | 1:200 |
| 2 | NF-kB (p65) | Cell Signaling Technology | 8242S | 1:400 |
| 3 | Ki-67 | Cell Signaling Technology | 12202P | 1:400 |
| 4 | CD8 | Cell Signaling Technology | 98941 | 1:400 |
| 5 | CD4 | Cell Signaling Technology | 25229 | 1:100 |
| 6 | Granzyme B | Cell Signaling Technology | 46890 | 1:125 |
| 7 | FoxP3 | Cell Signaling Technology | 12653 | 1:400 |
